# Artificial neural network, predictor variables and sensitivity threshold for DNA methylation-based age prediction using blood samples

**DOI:** 10.1038/s41598-021-81556-2

**Published:** 2021-01-18

**Authors:** Zhonghui Thong, Jolena Ying Ying Tan, Eileen Shuzhen Loo, Yu Wei Phua, Xavier Liang Shun Chan, Christopher Kiu-Choong Syn

**Affiliations:** DNA Profiling Laboratory, Biology Division, Health Sciences Authority, 11 Outram Road, Singapore, 169078 Singapore

**Keywords:** DNA methylation, Methylation analysis, DNA methylation

## Abstract

Regression models are often used to predict age of an individual based on methylation patterns. Artificial neural network (ANN) however was recently shown to be more accurate for age prediction. Additionally, the impact of ethnicity and sex on our previous regression model have not been studied. Furthermore, there is currently no age prediction study investigating the lower limit of input DNA at the bisulfite treatment stage prior to pyrosequencing. Herein, we evaluated both regression and ANN models, and the impact of ethnicity and sex on age prediction for 333 local blood samples using three loci on the pyrosequencing platform. Subsequently, we trained a one locus-based ANN model to reduce the amount of DNA used. We demonstrated that the ANN model has a higher accuracy of age prediction than the regression model. Additionally, we showed that ethnicity did not affect age prediction among local Chinese, Malays and Indians. Although the predicted age of males were marginally overestimated, sex did not impact the accuracy of age prediction. Lastly, we present a one locus, dual CpG model using 25 ng of input DNA that is sufficient for forensic age prediction. In conclusion, the two ANN models validated would be useful for age prediction to provide forensic intelligence leads.

## Introduction

In recent years, technological developments and advancements in biomedical sciences has propelled forensic genetics into a new era of DNA intelligence, one example being the ability to predict the chronological age of the donor of a body fluid sample recovered from a crime scene. This investigative lead on age can potentially assist the police in narrowing down the number of suspects for cases where the unknown donor cannot be identified from DNA direct matching or by a search of the DNA database. Previous molecular approaches for age prediction rely on shortening of telomeres^1,2^, mitochondrial DNA deletions^3,4^, advanced glycation end-products^5^, aspartic acid racemization^6,7^, and signal-joint T-cell receptor excision circles^8^. These approaches, however, have several limitations including large standard error, limited age range, inconsistent sampling procedure and sophisticated methodology^2,4,8–10^ that limit their applicability in the forensic context.

Over the past 10 years, DNA methylation has gained acceptance as the standard approach to predict age. Initial models for epigenetic age prediction were based on genome-wide array platforms^11–14^. Koch and Wagner^12^ developed an age model using five CpGs for 13 different tissue types, but with differences between chronological and predicted ages as large as 11 years. A more accurate model was subsequently developed by Horvath et al.^13^, using 353 CpGs to predict age in 51 different tissue types with an improved average accuracy of 2.9–3.6 years. Nevertheless, the predicted age in some tissue types deviated from the chronological age by more than ± 10 years, indicating that the prediction accuracy is tissue-dependent or that different markers may be needed for different tissues in order to achieve high accuracy levels. Hannum et al.^14^ was the first group to report an age model for blood samples using 71 CpGs with an average error of 3.9–4.9 years. They were also the first to report on the effects of auxiliary variables such as gender and body mass index on age prediction. Although the genome-wide array platform is essential during the discovery stage, it may not be applicable in the forensic context due to expensive methodology, longer processing time and requirement of large amount of input DNA.

The pyrosequencing assay has been successfully applied to quantitate DNA methylation levels of targeted CpGs. Weidner et al.^15^ built a multivariate regression model with mean absolute deviation (MAD) of 5.4 years using just three CpGs for blood samples. The model was subsequently validated with MAD of 4.5 years in the test data. Within the forensic community, Zbieć-Piekarska et al.^16^ developed a multivariate regression model with MADs of 5.0–5.7 years using two CpGs from *ELOVL2* for blood samples. To improve the accuracy of the model, the group retrained the model with five CpGs from *ELOVL2*, *C1orf132/MIR29B2C*, *FHL2*, *KLF14* and *TRIM59* to decrease the MADs to 3.4 years and 3.9 years in the training and test data, respectively^17^. Thereafter, several groups have reported using CpGs from *ELOVL2* along with other age-associated markers for forensic age prediction in blood^18–22^, teeth^18,23^, saliva^22,24^ and buccal swabs^22^. The methodologies adopted were also varied, covering pyrosequencing^18–20^, EpiTYPER^21,23^, SNaPshot^22^ and high resolution melting^24^. Results from these studies cemented the position of *ELOVL2* as the primary marker for age prediction. Further studies also revealed that *ELOVL2* CpGs consistently display age-related increase in DNA methylation across many tissue types, while CpGs from other age-associated markers are generally tissue-specific^25^. Interestingly, *ELOVL2* is not associated with age in semen. Using the SNaPshot assay, Lee et al.^26^ developed a multivariate model with three CpGs from *TTC7B*, *NOX4* and cg12837463 to predict age from semen samples with MADs of 4.2–5.4 years.

In the past three years, studies have emerged employing a combination of massively parallel sequencing (MPS) technology and artificial intelligence to understand epigenetic age signatures. Vidaki et al.^27^ compared a regression model (MAD = 4.6 years) using 23 CpGs with an artificial neural network (ANN) model (MAD = 3.3 years) using 16 CpGs in training data. The ANN model was subsequently validated with MAD of 4.4 years in test data. Naue et al.^28^ developed a random forest regression model with MADs of 3.2 years in training data and 3.1 years in test data using 13 CpGs in blood samples. These two groups further explored the implication of different machine learning models^29^ and different tissues^30^ on age prediction. Additionally, Aliferi et al.^29^ also demonstrated accurate age prediction using only 10 ng of input DNA on the MPS platform. Nevertheless, there is a lack of studies showing the limit of input DNA on the pyrosequencing platform for forensic age prediction.

Amongst the various models reported, the Zbieć-Piekarska model^17^ is perhaps the most well-known age prediction model for targeted bisulfite treatment and pyrosequencing. We have previously retrained the Zbieć-Piekarska model to suit the local population in Singapore^31^. The retraining was performed to resolve potential differences attributed to population-specific differential methylation patterns as well as to investigate the use of fewer CpGs (less than five) to predict age in the forensic context. The accuracy of our retrained regression model was reported with MADs of 3.3 years and 5.0 years in the training data and test data, respectively^31^, suggesting signs of overfitting. Although our local population comprised three major ethnic groups—Chinese, Malays and Indians, the effects of ethnicity on age prediction were not investigated in our previous study. In the present study, we re-determined the best age predictors prior to (1) comparison of prediction accuracy between ANN and regression models, (2) investigation on the effect of co-variables such as ethnicity and sex on age prediction and (3) determination of the minimum amount of input DNA required for bisulfite treatment and pyrosequencing to predict age.

## Materials and methods

### Sample source and DNA extraction

The study was conducted using 333 blood samples of Singapore Chinese, Malay and Indian individuals aged between 0 and 88 years. These blood samples were from previous crime cases retained in the laboratory with identity information anonymised with the exception of age, ethnicity and gender. DNA was extracted from whole blood using the Wizard Genomic DNA Purification Kit (Promega Corporation, Madison, WI, USA) or the Maxwell 16 LEV Blood DNA Kit (Promega Corporation) on the Maxwell 16 instrument following the manufacturer’s protocol. The extracted DNA was quantified using the Quantifiler Duo DNA Quantification kit (Applied Biosystems, Foster City, CA, USA) on the QS7 Real-Time PCR System (Applied Biosystems).

### Bisulfite conversion and pyrosequencing

Age-associated CpGs within *ELOVL2*, *TRIM59*, *KLF14* and *FHL2* were evaluated through DNA methylation analysis conducted using bisulfite treatment and pyrosequencing. Previously published PCR primers and sequencing primers^17^ were employed (Supplementary Table S1). A starting template of 500 ng of DNA was subjected to bisulfite conversion using the EpiTect Fast Bisulfite Conversion Kit (Qiagen, Hilden, Germany) following the manufacturer’s protocol. Based on an 80% DNA recovery rate after bisulfite conversion (personal communication with Qiagen’s technical support specialist), 20 ng of bisulfite-treated DNA were used for PCR amplification of each locus using the PyroMark PCR Kit (Qiagen). Amplifications were performed in a total volume of 25 µL, containing 0.2 µM of standard primer, 0.2 µM of biotinylated primer, 20 ng of DNA template, and PyroMark PCR Master Mix (which contained HotStarTaq DNA Polymerase, 1 × PyroMark PCR Master Buffer and dNTPs). The amplification programme comprised an initial denaturation step at 95 °C for 15 min, 45 PCR cycles of 94 °C for 30 s, 56 °C for 30 s and 72 °C for 30 s, followed by a final extension at 72 °C for 10 min. Following amplification, 10 µL of the biotinylated PCR product was immobilized on 1 µL of Streptavidin-coated Sepharose high-performance beads (GE Healthcare, Chicago, IL, USA) and annealed with 20 µL of 0.375 µM sequencing primer for 5 min at 80 °C on the PyroMark vacuum prep workstation (Qiagen). Pyrosequencing was subsequently performed using the PyroMark Q24 Advanced CpG Reagents (Qiagen) on the PyroMark Q24 Advanced instrument (Qiagen) following the manufacturer’s protocol. The generated pyrograms were analysed using the PyroMark Q24 Advanced Software (Qiagen) to obtain the DNA methylation levels of targeted CpGs. For reproducibility assessment, ten blood samples were put through the assay in duplicates starting from bisulfite treatment.

### Sensitivity testing

For sensitivity testing, varying amounts of input DNA (500 ng, 25 ng, 15 ng) were bisulfite converted as described above. Subsequently, 10 ng of bisulfite-treated DNA were amplified and subjected to pyrosequencing.

### Experiment design and statistical analysis

The dataset of 333 blood samples was randomized into training and test sets in a ratio of 3:2. The training data of 196 blood samples (106 Chinese, 45 Malays, 45 Indians; 141 males and 55 females) between 0 and 88 years of age was used to develop the age prediction model. Univariable regression analysis was applied to determine the correlation between DNA methylation level and chronological age. Forward stepwise regression was performed to simultaneously analyse the 29 CpGs from *ELOVL2*, *TRIM59*, *KLF14* and *FHL2* to identify suitable CpGs as predictors of chronological age. Beginning with an empty model, best CpG predictor was sequentially added in each iterative step based on a default entry of probability of F (0.05). The iteration stopped when no CpG predictor met the entry criterion.

The multivariable regression (MVR) equation developed based on the 196 training samples was validated using an independent set of 137 test samples (63 Chinese, 33 Malays, 41 Indians; 108 males and 29 females) with ages ranging from 0 to 85 years. The MVR formula used in this study was:

$$ {\text{Predicted}}\,{\text{age}}\,\left( {{\text{years}}} \right) \, = \, - {25}.{991 } + \, 0.{762} \times \, \left[ {ELOVL\mathit{2}\,{\text{C4}}} \right] \, + {1}.{162 } \times \, \left[ {KLF\mathit{14}{\text{C1}}} \right] \, + \, 0.{589 } \times \, \left[ {TRIM{\mathit{59}}{\text{C5}}} \right]. $$

Besides MVR, multilayer perceptron (MLP), which is a class of ANN, was also used to develop a model based on *ELOVL2* C4, *KLF14* C1 and *TRIM59* C5 using the same training data. Neural network module of IBM SPSS statistics was used to build the ANN model. The network architecture, which uses feedforward method, is composed of three layers: input layer, hidden layer and output layer. Before training, the data was randomly assigned to training (70%) and test (30%) subsets. The network was built with three inputs (*ELOVL2* C4, *KLF14* C1 and *TRIM59* C5), two units in hidden layer, and one output (Supplementary Fig. S1). One hidden layer and an automatically selected number of units (between 1 and 50) was applied. All covariates were normalized to values between 0 and 1. The data of age was rescaled as normalization in the range of 0 to 1 by correction of 0.02. The activation function linked the weighted sum units in a layer to the values of units in the succeeding layer. The hidden layer was activated by the hyperbolic tangent function, and the output layer was activated by the identity function. For the remaining settings, default parameters of the IBM SPSS statistics were applied (Supplementary Table S2). The synaptic weights (also referred to as coefficient estimates) that show the relationship between the units in a given layer to the units in the following layer (summarized in Supplementary Table S3), were exported as .xml file. These training iterations were repeated until the network adjusted the synaptic weights to produce predictions with only a minimal difference to the actual values. Subsequently, the trained ANN model was validated by applying the .xml file to the test set of 137 samples. For sensitivity testing, the ANN models were trained similarly as described, but with only one CpG (*ELOVL2* C4), or two CpGs (*ELOVL2* C4 and *ELOVL2* C5).

To evaluate the MVR and ANN models, mean absolute deviations (MADs) from chronological age were calculated for the training and test data. Additionally, prediction results were interpreted as correct if the predicted age was within ± 5 years of chronological age. This cut-off value was determined according to the root mean square error (RMSE) of the developed MVR model. To investigate the influence of different age groups on prediction accuracy, deviation of predicted age from chronological age was evaluated for four age groups: ‘Below 21’, ‘21–40’, ‘41–60’ and ‘61 and above’. Wilcoxon signed-rank test was used to assess the MADs to evaluate the difference between the MVR and ANN models for all age groups in both the training and test data. Kruskal–Wallis test followed by Dunn-Bonferroni post-hoc comparison was used to assess the deviations.

To explore the effects of ethnicity and sex on age prediction, deviations from chronological age were categorized according to the different ethnic groups and sex using the developed ANN model. DNA methylation levels of targeted CpGs for Polish and French populations reported by Zbieć-Piekarska et al.^17^ and Daunay et al.^32^ were used for further comparison with foreign ethnic groups. There was no further treatment to these reported data to eliminate potential technical variation prior to using them to evaluate our ANN model. Kruskal–Wallis test followed by Dunn–Bonferroni post-hoc comparison was used to assess the deviations for the different ethnic groups. Two-tailed t-test was used to assess the deviations for each sex.

For sensitivity testing, forward stepwise regression analysis was conducted on the 196 training samples data to determine the *ELOVL2* CpG predictors to be used in retraining of the ANN model. Subsequently, the ANN models were trained as described above. The ANN models developed were evaluated on 27 individuals (11 Chinese, 10 Malays, 6 Indians) where methylation data were obtained using 500 ng, 25 ng and 15 ng of input DNA for bisulfite conversion.

All analyses were performed using IBM SPSS statistics ver. 25. Shapiro–Wilk test was conducted to assess for data normality prior to application of a statistical test. An alpha of 0.05 was used as the cut-off for significance for all analyses involved in this study. The .xml files for ANN models and step-by-step instructions are provided in the supplementary information.

## Results

### Reproducibility assessment

To assess the reproducibility of the pyrosequencing assay, 10 blood samples were separately bisulfite-converted and amplified prior to pyrosequencing for all four genes (*ELOVL2*, *TRIM59*, *KLF14* and *FHL2*). The mean difference in DNA methylation between conversions for all the 29 CpGs was below 3% (Supplementary Table S4), comparable to that reported by Tost and Gut even in cases of different bisulfite treatments and/or separate PCR reaction^33^, indicating that our assays were highly reproducible.

### Correlation analysis of age-associated markers

To reconfirm the magnitude of age association for the CpGs in *ELOVL2*, *TRIM59*, *KLF14* and *FHL2*, bisulfite treatment and pyrosequencing were used to quantitate the DNA methylation levels of the 29 CpGs using 196 blood samples. Strong positive correlations (0.731 ≤ *R* ≤ 0.947) between methylation levels and age were observed for all examined CpGs (Table 1, Supplementary Fig. S2). The correlation analysis also revealed significant associations (*P* < 0.001) for all examined CpGs (Table 1). The strongest correlation for each locus was observed at *ELOVL2* C4 (*R* = 0.947), *KLF4* C1 (*R* = 0.860), *TRIM59* C5 (*R* = 0.919) and *FHL2* C1 (*R* = 0.932), explaining 89.6%, 73.8%, 84.4% and 86.7% of age-associated variation, respectively. These results indicated that changes in DNA methylation levels in these four loci are highly associated with aging.

**Table 1:** Univariable regression analysis and chromosomal coordinates of 29 CpGs at four DNA methylation loci using 196 training data. Map coordinates refer to the genomic positions in human reference genome 38 (GRCh38).

Locus	CpG	Chr	Map info (GRCh38)	*R*	Adjusted *R*^2^	Standard error	*P* value
*ELOVL2*	C1	6	11044661	0.907	0.822	9.020	9.72 × 10^–75^
	C2	6	11044655	0.905	0.818	9.107	6.35 × 10^–74^
	C3	6	11044647	0.920	0.845	8.397	9.00 × 10^–81^
	**C4**	**6**	**11044644**	**0.947**	**0.896**	**6.900**	**2.47** × **10**^**–97**^
	C5	6	11044642	0.946	0.894	6.956	1.19 × 10^–96^
	C6	6	11044640	0.945	0.893	6.990	3.08 × 10^–96^
	C7	6	11044634	0.913	0.833	8.736	1.93 × 10^–77^
*KLF14*	**C1**	**7**	**130734355**	**0.860**	**0.738**	**10.920**	**1.29** × **10**^**–58**^
	C2	7	130734357	0.817	0.666	12.339	2.69 × 10^–48^
	C3	7	130734373	0.824	0.678	12.123	8.70 × 10^–50^
	C4	7	130734375	0.749	0.559	14.178	1.49 × 10^–36^
*TRIM59*	C1	3	160450172	0.851	0.723	11.229	2.94 × 10^–56^
	C2	3	160450174	0.821	0.672	12.218	4.00 × 10^–49^
	C3	3	160450179	0.907	0.821	9.035	1.34 × 10^–74^
	C4	3	160450184	0.879	0.772	10.201	2.33 × 10^–64^
	**C5**	**3**	**160450189**	**0.919**	**0.844**	**8.425**	**1.70** × **10**^**–80**^
	C6	3	160450192	0.916	0.838	8.582	6.23 × 10^–79^
	C7	3	160450199	0.913	0.834	8.711	1.10 × 10^–77^
	C8	3	160450202	0.898	0.805	9.438	6.49 × 10^–71^
*FHL2*	**C1**	**2**	**105399282**	**0.932**	**0.867**	**7.775**	**2.90** × **10**^**–87**^
	C2	2	105399288	0.903	0.815	9.181	3.00 × 10^–73^
	C3	2	105399291	0.907	0.821	9.021	9.88 × 10^–75^
	C4	2	105399297	0.833	0.693	11.828	7.28 × 10^–52^
	C5	2	105399300	0.887	0.785	9.901	7.03 × 10^–67^
	C6	2	105399310	0.847	0.716	11.377	3.79 × 10^–55^
	C7	2	105399314	0.818	0.667	12.319	1.96 × 10^–48^
	C8	2	105399316	0.821	0.672	12.225	4.46 × 10^–49^
	C9	2	105399323	0.808	0.651	12.614	1.97 × 10^–46^
	C10	2	105399327	0.731	0.531	14.615	5.49 × 10^–34^

CpG from each locus with the strongest correlation with age is highlighted in bold.

*Chr* chromosome.

### Retraining of age prediction model

To improve on the accuracy and resolve potential overfitting in our previous age prediction model, we randomized the 333 samples into a training (n = 196) and test set (n = 137). Forward stepwise regression analysis was performed on the 29 CpGs for the 196 training samples to establish a suitable age prediction model comprising the most informative CpG predictors. In this iterative process, CpG predictors were added sequentially into the model to assess the statistical improvement (if any) to the model. As shown in Supplementary Table S5, the model with *ELOVL2* C4 alone was observed with RMSE of 6.9 years and MAD of 5.2 years, accounting for 89.6% of age-associated variation (95% CI 0.869–0.923). With the addition of *KLF14* C1 and *TRIM59* C5, the model showed an improved age predictive power with RMSE of 5.4 years and MAD of 4.1 years, explaining 93.7% of age-associated variation (95% CI 0.922–0.954). With more than three predictors, confidence intervals of the *R*^2^ were largely overlapping. Furthermore, models with four and five predictors had variance inflation factor (VIF) values of more than 10. A VIF value above 10 indicates multicollinearity among the predictors, which could potentially inflate the variance of estimates until the model is rendered unstable^34^. As such, *ELOVL2* C4, *KLF14* C1 and *TRIM59* C5 were selected as the age predictors for building the multivariable regression (MVR) and ANN models.

### Applying artificial neural network for age prediction

**Figure 1:**
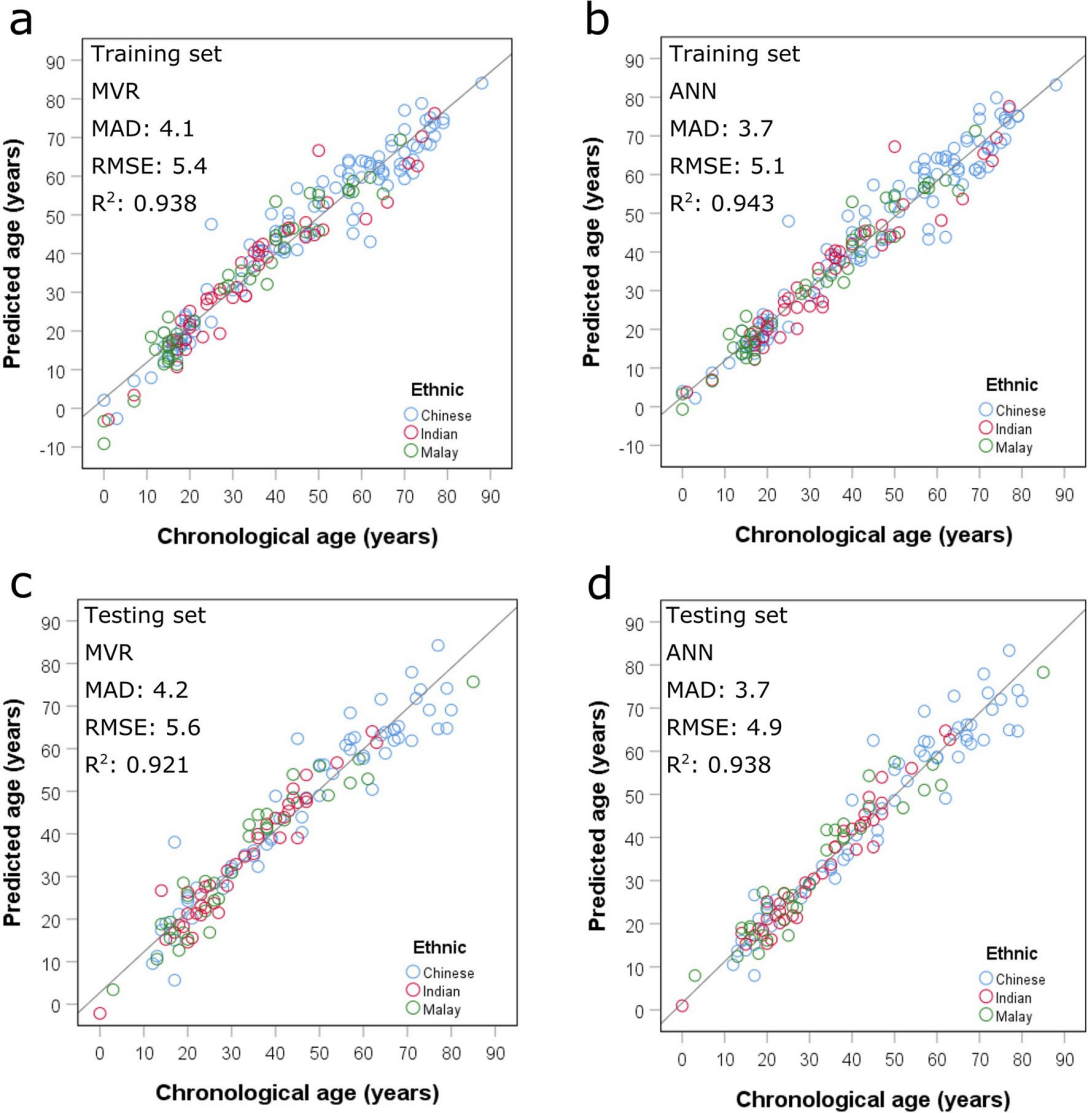
Age prediction with multivariable regression (MVR) and artificial neural network (ANN) models on (**a**,**b**) training (n = 196) and (**c**,**d**) test (n = 137) data comprising the three local ethnic groups. Age was predicted with three predictors—*ELOVL2* C4, *KLF14* C1 and *TRIM59* C5. *MAD* mean absolute deviation; *RMSE* root mean square error. The units for MAD and RMSE are years.

To examine whether machine learning can predict chronological age more accurately, we developed an ANN model using the three selected age predictors with the same training data and compared its predictive performance with the MVR model. Although confidence intervals of the age-associated variation explained by the ANN and MVR models were largely overlapping (Supplementary Table S5), the ANN model had higher accuracy with MAD of 3.7 years, which was significantly different from that of the MVR model with MAD of 4.1 years (*P* = 0.001, Fig. 1a,b, Table 2). The ANN model also displayed a higher percentage of correct prediction (75.5%) when compared with the MVR model (70.9%). Among the different age groups, the ANN model had significantly lower MADs than the MVR model in two age groups—‘Below 21’ (MADs = 2.2 vs 2.9 years, *P* = 0.001) and ‘21–40’ (MADs = 3.5 vs 4.1 years, *P* = 0.005). As expected, these two younger age groups also exhibited a higher percentage of correct prediction with the ANN model (92.6%, 80.0%, respectively) than with MVR model (79.6%, 74.0%, respectively). No difference in MADs (*P* > 0.05) were observed for both models with respect to the two older age groups—‘41–60’ and ‘61 and above’. These data suggested that the ANN model overall could predict the age of an individual more accurately than the MVR model.

**Table 2:** MADs and percentage of correct predictions of multivariable regression (MVR) and artificial neural network (ANN) models for different age groups with both training and test data.

Data	Age group	MAD (years)			% Correct prediction (n)	
		MVR	ANN	*P* value	MVR	ANN
Training	Below 21	2.9	2.2	0.001***	79.6 (43/54)	92.6 (50/54)
	21–40	4.1	3.5	0.005**	74.0 (37/50)	80.0 (40/50)
	41–60	4.1	4.2	0.951	70.8 (34/48)	68.8 (33/48)
	61 and above	5.3	5.2	0.949	56.8 (25/44)	56.8 (25/44)
	Overall	4.1	3.7	0.001***	70.9 (139/196)	75.5 (148/196)
Test	Below 21	4.3	3.1	0.001***	73.3 (22/30)	86.7 (26/30)
	21–40	2.9	2.6	0.130	80.9 (38/47)	85.1 (40/47)
	41–60	4.2	4.1	0.422	65.7 (23/35)	62.9 (22/35)
	61 and above	6.3	5.8	0.326	40.0 (10/25)	48.0 (12/25)
	Overall	4.2	3.7	0.002**	67.9 (93/137)	73.0 (100/137)

Correct prediction was calculated based on ≤ 5 years between predicted and actual ages. Asterisk denotes the degree of significance.

*MAD* mean absolute deviation.

### Validation of age prediction models

To validate the accuracy of the MVR and ANN models for age prediction and determine if these models were overfitting, we challenged the models with another independent set of 137 blood samples. The ANN model displayed no difference in prediction accuracy (MADs = 3.7 years) between the training and test data (Fig. 1b,d) while the MVR model exhibited only a small difference in accuracy (MADs = 4.1 vs 4.2 years) between the datasets (Fig. 1a,c). These results indicated the absence of overfitting in both the MVR and ANN models. In concordance with the training data, the ANN model was validated with a higher accuracy when compared with the MVR model (MADs = 3.7 vs 4.2 years, *P* = 0.002, Table 2). The ANN model also produced a higher percentage of correct prediction (73.0%) for predicted age when compared with the MVR model (67.9%), which was consistent with the training data. Among the different age groups, the ANN model had a significantly lower MAD than the MVR model for the youngest age group—‘Below 21’ (MADs = 3.1 vs 4.3 years, *P* = 0.001). The results showed that the ANN model outperformed the MVR model in predicting the chronological age of an individual, especially for the younger individuals.

### Effect of different age groups on age prediction

**Figure 2:**
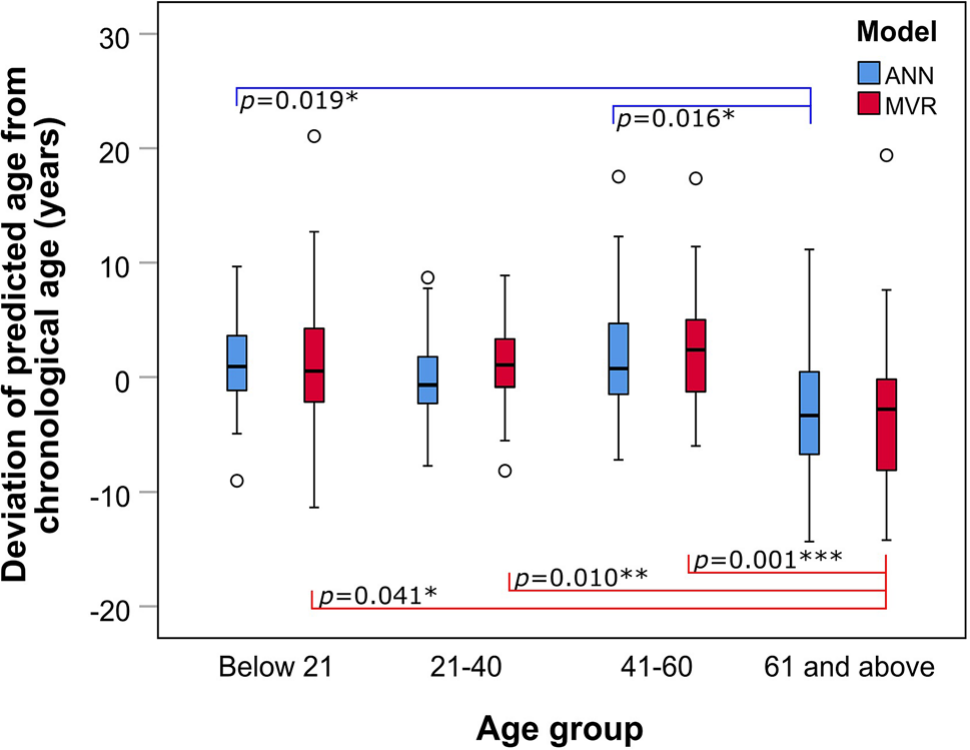
Boxplot showing deviation of predicted age from chronological age for 137 blood samples determined by multivariable regression (MVR) and artificial neural network (ANN) models for the different age groups. All pairwise comparisons with statistical significance (*P* < 0.05) are indicated. Asterisk denotes the degree of significance. Open circle denotes outlier.

MADs in the training data were observed to generally increase from the youngest age group (2.2–2.9 years) to the oldest age group (5.2–5.3 years), although this increasing trend was less prominent in the test data (Table 2). To explore whether the increasing deviation of predicted age from chronological age correlates with chronological age, we plotted the deviations against chronological age. The Blant–Altman plots did not reveal any obvious increase in deviations as age increases (Supplementary Fig. S3). The age of older individuals however could be observed to be underestimated. This underestimation of age for the older individuals was most apparent when the deviations were categorized according to the four different age groups (Fig. 2). The deviations from chronological age obtained using the MVR model showed significant difference between the age group ‘61 and above’ and the other age groups (*P* = 0.041 for ‘Below 21’; *P* = 0.010 for ‘21–40’; *P* = 0.001 for ‘41–60’). Additionally, significant differences between the age group ‘61 and above’ and two other age groups (*P* = 0.019 for ‘Below 21’; *P* = 0.016 for ‘41–60’) were also observed for the ANN model.

### Variables affecting age prediction

**Figure 3:**
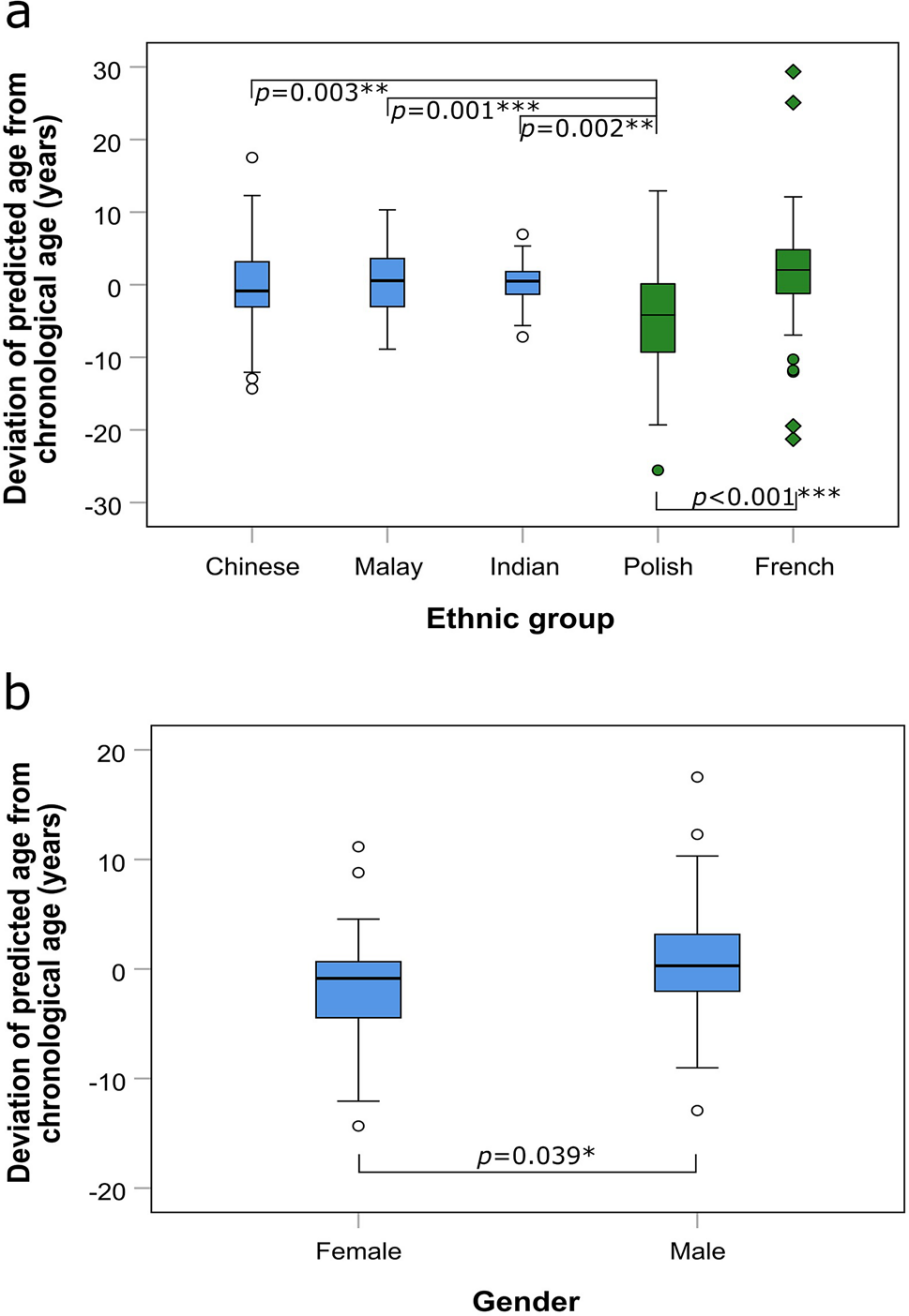
Boxplot showing deviation of predicted age from chronological age determined by the artificial neural network (ANN) model for the different (**a**) ethnic groups and (**b**) sex. For ethnicity analysis, the dataset consisted of 63 Chinese, 33 Malays, 41 Indians, 120 Polish and 100 French. For sex analysis, the dataset consisted of 108 males and 29 females from the local Chinese, Malay and Indian populations. All pairwise comparisons with statistical significance (*P* < 0.05) were indicated. Asterisk denotes the degree of significance. Circle denotes outlier. Diamond denotes extreme outlier.

To determine whether co-variables such as ethnicity affect age prediction, the deviations from chronological age was examined for the three ethnic groups in our local population using the ANN model. No significant difference (*P* = 0.531) in deviations was observed among the Chinese, Malays and Indians using the ANN model (Fig. 3a). A significant difference, however, was observed for the Polish when compared with the other ethnic groups including the French (*P* ≤ 0.003). An underestimation of age was generally observed for the Polish samples while extreme outliers were observed for the French samples on our ANN model. These data indicated that prediction accuracy using our ANN model would be lower for the Polish and French populations, while having no impact on age prediction among the local Chinese, Malay and Indian populations. Therefore, our ANN model is better suited for the local ethnic groups in Singapore.

To examine whether sex affects age prediction, the deviations from chronological age was examined by sex in our local population. Although the predicted ages of men were marginally higher than that for women (*P* = 0.039, Fig. 3b), sex does not have an effect on age prediction.

To confirm the effect of ethnicity and sex on age prediction for our local population, we developed MVR models holding ethnicity and/or sex constant. Our default MVR model provided an accuracy with MADs of 4.1–4.2 years (Fig. 1; Table 2). With an adjustment for ethnicity and/or sex, there was no prominent improvement in prediction accuracy observed (MADs = 4.0–4.2 years) (Supplementary Table S6). Using a cut-off of more than 10% change in the regression coefficient to identify any confounding factors, we observed less than a 10% change for all three predictors adjusted for ethnicity and/or sex (Supplementary Table S7). The data demonstrated that the accuracy of our age prediction model is unlikely to be affected by local ethnicity and/or sex.

### Sensitivity testing

As multiplexing is not readily achievable during pyrosequencing, larger quantities of DNA template are required when more loci are to be analysed for DNA methylation. To reduce the amount of input DNA used for age prediction, forward stepwise regression was performed to identify the most informative *ELOVL2* CpGs that can be used to build an ANN model. The dual CpG model comprising C4 and C5 was observed with RMSE of 6.1 years (Supplementary Table S8), which was lower than the single CpG model comprising C4 with RMSE of 6.9 years (also see Supplementary Table S5). Notably, the dual CpG model had high VIFs of 10 for both CpGs, a value which was the upper limit of acceptable multicollinearity^34^. The presence of multicollinearity was likely due to the close proximity of both CpGs in their chromosomal positions. With the addition of C1 to the dual CpG model, a higher degree of multicollinearity (VIF = 16.021) was detected with no change in RMSE. Therefore, we focused on evaluating the single and dual CpG sensitivity ANN models. Using 500 ng of input DNA, the dual CpG model exhibited a relatively better performance (MADs = 4.4–4.7 years) when compared with the single CpG model (MADs = 4.7 years) (Supplementary Fig. S4). Nevertheless, the dual CpG model still has a lower accuracy for age prediction when compared with the default ANN model with three CpGs from three loci (MADs = 3.7 years, Fig. 1b,d; Table 2).

To determine the minimum amount of input DNA required for age prediction with only one locus *ELOVL2*, two different amounts of input DNA (25 ng and 15 ng) for bisulfite conversion were evaluated. These two amounts were decided by taking into consideration the 80% DNA recovery from bisulfite conversion as well as the minimum requirement of 10 ng of bisulfite-treated DNA for reproducible amplification^35^. The bisulfite pyrosequencing with 15 ng of input DNA, however, produced either no or low pyrosequencing signal. Thus, the evaluation of sensitivity was based on the original amount (500 ng) and 25 ng of input DNA.

Based on 500 ng of input DNA on 27 blood samples, the single and dual CpG ANN models (MADs = 3.2 and 3.1 years, respectively) had comparable prediction accuracies as that of the default ANN model comprising three CpGs from three loci (reference MAD = 3.2 years, Table 3). When both sensitivity ANN models were challenged with the reduced input DNA quantity of 25 ng, the accuracies of the single and dual CpG models decreased (MADs = 3.7 and 3.5 years, respectively). Our results showed that the dual CpG model performed marginally better than the single CpG model, especially with 25 ng of input DNA. The architecture of the dual CpG sensitivity ANN model and its parameter estimates are shown in Supplementary Fig. S5 and Table S9, respectively.

**Table 3:** MADs and percentage of correct predictions of different artificial neural network (ANN) models using varying amounts of input DNA for age prediction on 27 local individuals.

Model	Input DNA (ng)	MAD (years)	Correct prediction % (n)
Default ANN	500	3.2	81.5 (22/27)
Single CpG	500	3.2	77.8 (21/27)
	25	3.7	85.2 (23/27)
Dual CpG	500	3.1	85.2 (23/27)
	25	3.5	88.9 (24/27)

Default ANN = original ANN model comprising three CpGs from *ELOVL2* C4, *KLF14* C1 and *TRIM59* C5. Single CpG = sensitivity ANN model comprising only *ELOVL2* C4. Dual CpG = sensitivity ANN model comprising *ELOVL2* C4 and C5. Correct prediction is calculated based on ≤ 5 years between predicted and actual ages. *MAD* mean absolute deviation.

## Discussion

The regression-based age prediction model developed in our previous study was recently independently evaluated to be a better performing model for the French population^32^. However, we have attempted to further retrain our model as the 1.7 years difference in MADs obtained from our previous training (MAD = 3.3 years) and test (MAD = 5.0 years) data^31^ suggested possible overfitting. In the current training data, *ELOVL2* C4 was observed to exhibit the strongest correlation with age (Table 1) as opposed to C5 in our previous study^31^. Notably, the top three *ELOVL2* CpGs were consistently C4–C6 and their differences in correlation strength were negligible. Similarly, negligible differences in age correlation among the top three *TRIM59* CpGs (C5–C7) were observed. *KLF14* C1 and *FHL2* C1 also concordantly displayed the strongest correlation with age in both studies. In comparison to the *R* coefficients observed in the Zbieć-Piekarska et al. study^17^, several *R* coefficients observed in our previous study^31^ and present study exceeded 0.9. The higher *R* coefficients in our study could be attributed to the larger age range used in our training model (0–88 years) as compared with the Zbieć-Piekarska et al. study (2–75 years). This is consistent with the report by Daunay et al.^32^ that a smaller age range was responsible for the lower *R* coefficients observed in their study. These results suggested that *R* coefficient is highly dependent on the age range of samples used. As compared to the MVR model developed in our previous study^31^, the retrained MVR model comprised *ELOVL2* C4 (replacing C5), *KLF14* C1 (replacing C2) and *TRIM59* C5. The current MVR model had a higher precision (MADs = 4.1–4.2 years, Fig. 1a,c and Table 2) when compared with the previous model (MADs = 3.3–5.0 years)^31^. Importantly, the 0.1 year difference in MADs between the training and test data indicated the absence of overfitting in the current MVR model. Although the current model produced a higher MAD of 4.1 years in the training data, we believe it is more robust and reliable than the previous model^31^. Moreover, an improvement in prediction accuracy over the previous MVR model^31^ was also observed when the current MVR model was used to evaluate the French samples—a decrease from the reported MAD of 5.2 years^32^ to MAD of 5.0 years (Supplementary Table S10). Similarly, French samples with age predicted correctly had also increased from the reported 55^32^–61% (Supplementary Table S10). Together, these results demonstrated that the current MVR model performed better than our previous MVR model^31^ for age prediction.

In recent years, artificial intelligence (AI) has been increasingly applied towards predictive analytics in biomedical research. Machine learning, which is a subset of AI, constructs algorithms that can learn from data and make predictions. One such example is that of the artificial neural network (ANN, which comprises of layers of neurons that interact via carefully weighted connections to produce a predictable outcome. Due to its adaptive learning process, ANN reduces errors in prediction outputs by systematically optimising the connecting weights between the neurons within the network. Recent studies have demonstrated that age prediction using ANN models gave higher prediction accuracies than regression models^27,29,36,37^. In our present study, we observed a similar higher age prediction accuracy using an ANN model over a MVR model in both training (MADs = 3.7 vs 4.1 years, *P* = 0.001) and test data (MADs = 3.7 vs 4.2 years, *P* = 0.002) (Fig. 1; Table 2). Using the ANN model, we also obtained a higher prediction accuracy for the French samples (MAD = 4.8 years, 68% correct prediction), exceeding the accuracy obtained by the current MVR model (MAD = 5.0 years, 61% correct prediction) (Supplementary Table S10).

From a forensic casework perspective, both MVR and ANN models work well and can be applied towards predicting the age of the donor of the blood sample. However, the MVR model has a lower accuracy for overall age prediction. The MVR model works best with straight-line relationships, and attempts to determine the best-fit line for non-linear trends. In contrast, the innate ability of the ANN model to learn holistically from the observed non-linear trends (Supplementary Fig. S2), accounts for higher accuracy. One major caveat of using ANN model is overfitting where a model with high variance is made to achieve higher accuracy. Overfitting can also be caused by excessive input variables, insufficient training samples, or complicated ANN structures that use several hidden layers that consequently result in poor generalization. These overfitting issues were, however, not observed in the present study, as demonstrated by the similar MAD values obtained for the training and test sets.

Although MAD is usually used to assess the overall accuracy of the prediction model, it may be an over-simplification as prediction error has been observed to increase with age^38^. We therefore divided our data into four distinct age groups for further analysis on the prediction accuracy. The age boundary of each age group was set based on the age demographics of the local convicted offender population^39^ with slight modifications (Supplementary Table S11). It was observed that MADs generally increased from the youngest age group to the oldest age group (Table 2), which was consistent with observations in other studies^17–19^. In principle, MAD measures the average of all deviations from true age, but it lacks the capability to measure the spread of the deviations. By assessing the deviations from chronological age for every sample, we observed larger deviations for samples above 61 years of age. (Supplementary Fig. S3). This was further illustrated when the deviations from chronological age were categorized according to the different age groups (Fig. 2). The median lines from age group “61 and above” were observed to lie below other boxes belonging to the three other age groups, indicating the elderly was underestimated for age prediction. In contrast, the interquartile ranges of boxplots belonging to the three other age groups were largely overlapping, suggesting no difference in the deviations. The underestimation of age for the elderly could be attributed to an epigenetic drift in which the DNA methylation pattern altered due to more environmental stress experienced by the elderly. Together, our results indicated there is no difference in prediction accuracy for individuals below age 61. However, age prediction of individuals aged 61 and above are likely to be less accurate.

As intergroup variability such as ethnically diverse population and gender could be associated with variation in epigenetic age^38^, it is important to evaluate these effects on age prediction accuracy. In the present study, no significant difference in prediction error (*P* = 0.531) was observed among the local Chinese, Malays and Indians (Fig. 3a). This observation could be attributed to the incorporation of the three ethnic groups in the training data, thus accounting for most of the variations in DNA methylation patterns due to ethnic differences. This may also explain why there was no obvious change in prediction accuracy when ethnicity with and without sex was adjusted (Supplementary Table S7). However, pronounced variations in prediction errors were observed when the ANN model was used on the Polish sample population (Fig. 3a). The Polish sample was underestimated and its prediction error was significantly different (*P* ≤ 0.003) compared to all ethnic groups investigated in the present study. Despite the notable differences in prediction accuracy observed for the Polish and French samples, it may be possible that these differences could, in part, be due to methodology and instrumental variations during bisulfite conversion and/or pyrosequencing. As such, the ethnic effects from foreign populations may not be conclusive. We would suggest that a model trained with targeted ethnic groups should not be applied to an individual from a non-targeted ethnic group without model retraining. As our model comprised all three local ethnic groups, further study could be performed to directly evaluate the influence of ethnic-specific models on other ethnic groups. But for practical reasons, we were of the opinion that ethnic-specific model for each ethnic group in our local population may be less helpful for law enforcement as confusion may arise with separate models, for example, 30 ± 3.7 years old for Chinese, 32 ± 4.3 years for Malay, or 29 ± 4.1 years old for Indian. This was further supported by our finding that there was no prominent difference in prediction accuracy among the three ethnicities. Therefore, we sought to optimise a single model with a sufficiently high level of accuracy to the three ethnic groups in our population to be more practical for crime investigation.

For the effect of sex on age prediction, the results showed that there was marginal overestimation for males as compared to females (*P* = 0.039, Fig. 3b), similar to that observed in studies by Weidner et al.^15^, Zbieć-Piekarska et al.^17^ and Naue et al.^28^. It should be noted that there were studies which had suggested that gender had no effect on age prediction^12,18,21,27,32^. In the present study, there was no apparent change in prediction accuracy even when sex with and without ethnicity was adjusted (Supplementary Table S7). This observation could possibly be due to the weaker contribution by sex, which was consistent with the findings by Zbieć-Piekarska et al.^17^ and Naue et al.^28^. Likewise, although the DNA methylome in men “ages” faster (~ 4%) than in women, there was no difference in aging between the two genders^14^. Together, our results showed that sex has no impact on age prediction.

The age prediction models discussed thus far were evaluated based on three loci predictors—*ELOVL2* C5, *KLF14* C1 and *TRIM59* C5. A considerable amount of DNA is required to predict age using the three predictor model as singleplex amplification reactions had to be performed prior to pyrosequencing. This approach may not be feasible in the forensic context where crime evidential material may often be limiting. We therefore explored the feasibility of an alternative age prediction model that predicts age with just a single predictor *ELOVL2*. To obtain a higher accuracy for age prediction, we evaluated models that included more than one *ELOVL2* CpG while taking into account the multicollinearity effects on age prediction. Our multicollinearity diagnostic supported the inclusion of up to two *ELOVL2* CpGs in the model, *ELOVL2* C4 and C5 (Supplementary Table S8). Zbieć-Piekarska et al.^16^ also reported a dual *ELOVL2* CpG model, though their model comprised *ELOVL2* C5 and C7. Although the ANN model comprising two *ELOVL2* CpGs had a relatively higher accuracy (MADs = 4.4–4.7 years) than the ANN model with a single *ELOVL2* CpG (MADs = 4.7 years) (Supplementary Fig. S4), its predictive performance was still lower as compared with the MVR (MADs = 4.1–4.2 years) and ANN (MADs = 3.7 years) models that comprised of three loci predictors (Fig. 1). Nevertheless, the dual *ELOVL2* CpG model may be of practical relevance during forensic casework applications when only a limited amount of DNA is available for age prediction assays.

With the development of the one-locus *ELOVL2* model, we tested the performance of the model with a reduced amount of input DNA used for bisulfite conversion on 27 blood samples. With an input DNA of 500 ng, both the single and dual *ELOVL2* CpG models have comparable prediction accuracies (MADs = 3.2 and 3.1 years, respectively) as that of the default model with three CpGs from three loci (MAD = 3.2 years) (Table 3). This observation could be attributed to the smaller sample size examined. With an input DNA of 15 ng, we were unable to obtain reliable pyrosequencing results (due to no/low pyrosequencing signal), likely due to insufficient amounts of bisulfite-treated DNA being used for amplification. We postulated that the DNA recovery rate after bisufilte conversion decreases as the amount of input DNA decreases. As there is currently no standard approach to accurately quantify bisulfite-treated DNA, most studies to date have relied on theoretical recovery rates proposed by manufacturers. As such, the reported amount of bisulfite-treated DNA is generally perceived as arbitrary. While the recovery rate of bisulfite-treated DNA does not have a significant impact when larger amounts of input DNA are used (ie. at least 50 ng DNA), it can affect studies involving lower amounts of input DNA. It is therefore useful that methods to accurately quantify bisulfite-converted DNA be developed so as to maximise the potential of epigenetic applications in the forensic context.

Using a slightly higher amount of input DNA of 25 ng, we were able to obtain good quality pyrosequencing results. We observed that the dual CpG model performed better than the single CpG model (MADs = 3.5 vs 3.7 years). While a recent MPS-based study had demonstrated that 10 ng of input DNA was sufficient to produce an accurate age prediction^29^, the MPS approach has a longer preparation and processing time compared to pyrosequencing. Furthermore, it has been reported that MPS may be less accurate compared with pyrosequencing for epigenetic age prediction^40^. Though a previous pyrosequencing study first reported using only 10 ng of DNA on two *ELOVL2* CpGs without notable change in prediction error^16^, this value refers to the bisulfite-treated, PCR-ready DNA rather than the initial amount of input DNA used for bisulfite conversion, that would determine the minimal amount of DNA required for age prediction. Therefore, this may be the first study to demonstrate the minimal amount of DNA to perform bisulfite conversion followed by pyrosequencing for age prediction.

## Conclusion

Our study has demonstrated that the use of an artificial neural network machine learning outperforms the conventional regression model in predicting age through quantitating the methylation levels of *ELOVL2* C4, *KLF14* C1 and *TRIM59* C5 on the pyrosequencing platform. There were no prominent differences in prediction error with increasing age, though the age of older individuals was observed to be underestimated. We also showed that ethnicity did not affect the accuracy of our prediction model when applied on our local Chinese, Malay and Indian populations, although the accuracy of age prediction may decrease if the model is used to predict for an individual from another ethnic population. Although the age of males was generally overestimated, the sex effect did not have an impact on the accuracy of age prediction. Lastly, our study also reported a dual CpG model based on only the *ELOVL2* locus which could be used to predict age with as little as 25 ng of input DNA for bisulfite conversion followed by pyrosequencing. This may be of particular relevance in the forensic context when DNA evidence is often limited. We anticipate that our two validated ANN prediction models could be applied to predict the age of the donor of a sample as a forensic intelligence lead to help law enforcement officers narrow the pool of possible suspects.

## Supplementary Information

Supplementary Information.

Supplementary Information.

Supplementary Information.

Supplementary Information.

Supplementary Information.

Supplementary Information.
