# Author Correction: Artificial neural network, predictor variables and sensitivity threshold for DNA methylation-based age prediction using blood samples

**DOI:** 10.1038/s41598-021-88188-6

**Published:** 2021-04-13

**Authors:** Zhonghui Thong, Jolena Ying Ying Tan, Eileen Shuzhen Loo, Yu Wei Phua, Xavier Liang Shun Chan, Christopher Kiu-Choong Syn

**Affiliations:** DNA Profiling Laboratory, Biology Division, Health Sciences Authority, 11 Outram Road, Singapore, 169078 Singapore

Correction to: *Scientific Reports* 10.1038/s41598-021-81556-2, published online 18 January 2021.

The original version of this Article contained an error in the affiliation, which was incorrectly given as ‘DNA Profiling Laboratory, Applied Sciences Group, Health Sciences Authority, 11 Outram Road, Singapore 169078 Singapore’. The correct affiliation is listed below:

DNA Profiling Laboratory, Biology Division, Health Sciences Authority, 11 Outram Road, Singapore 169078, Singapore.

This error has now been corrected in the HTML and PDF versions of the Article, and in the accompanying Supplementary Information file.

