# Supplementary material for: Artificial neural network, predictor variables and sensitivity threshold for DNA methylation-based age prediction using blood samples
Source: Sci Rep. 2021 Jan 18;11:1744. doi: 10.1038/s41598-021-81556-2 (PMC7814006; doi:10.1038/s41598-021-81556-2)
Supplement: Supplementary file 1 — Supplementary Information. [file 41598_2021_81556_MOESM1_ESM.docx]

**Supplementary Information**

**Title**

Artificial neural network, predictor variables and sensitivity threshold for DNA methylation-based age prediction using blood samples

**Authors**

Zhonghui Thong^1^*, Jolena Ying Ying Tan^1^, Eileen Shuzhen Loo^1^, Yu Wei Phua^1^, Xavier Liang Shun Chan^1^, Christopher Kiu-Choong Syn^1^

**Affiliation and address of the authors**

^1^DNA Profiling Laboratory, Biology Division, Health Sciences Authority, 11 Outram Road, Singapore 169078, Singapore

***Email address and telephone of corresponding author**

[thong_zhonghui@hsa.gov.sg](mailto:thong_zhonghui@hsa.gov.sg)

+65 6213 0779 (telephone)


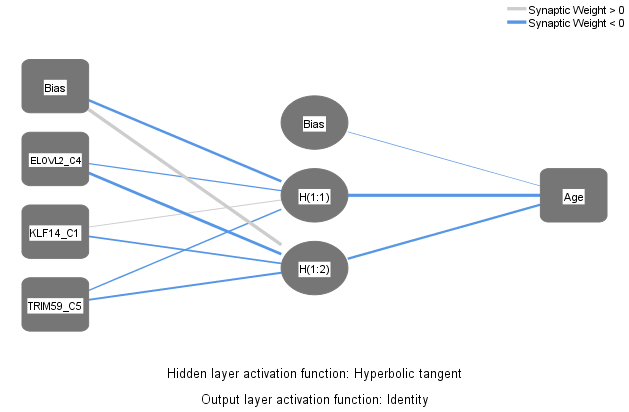


**Supplementary Figure S1**

Structural diagram of the artificial neural network (ANN) model. *ELOVL2* C4, *KLF14* C1 and *TRIM59* C5 are the input variables. All weighted values passed to the hidden layer node are summed on the hidden layer node and passed to the output node through activation function using hyperbolic tangent. All weighted values entering the output node are summed again and passed through activation function using identity. The output values (predicted age) in the training of the ANN are compared with the chronological age of each sample. The weight is adjusted closer to the chronological age the next time the sample appears on the network.


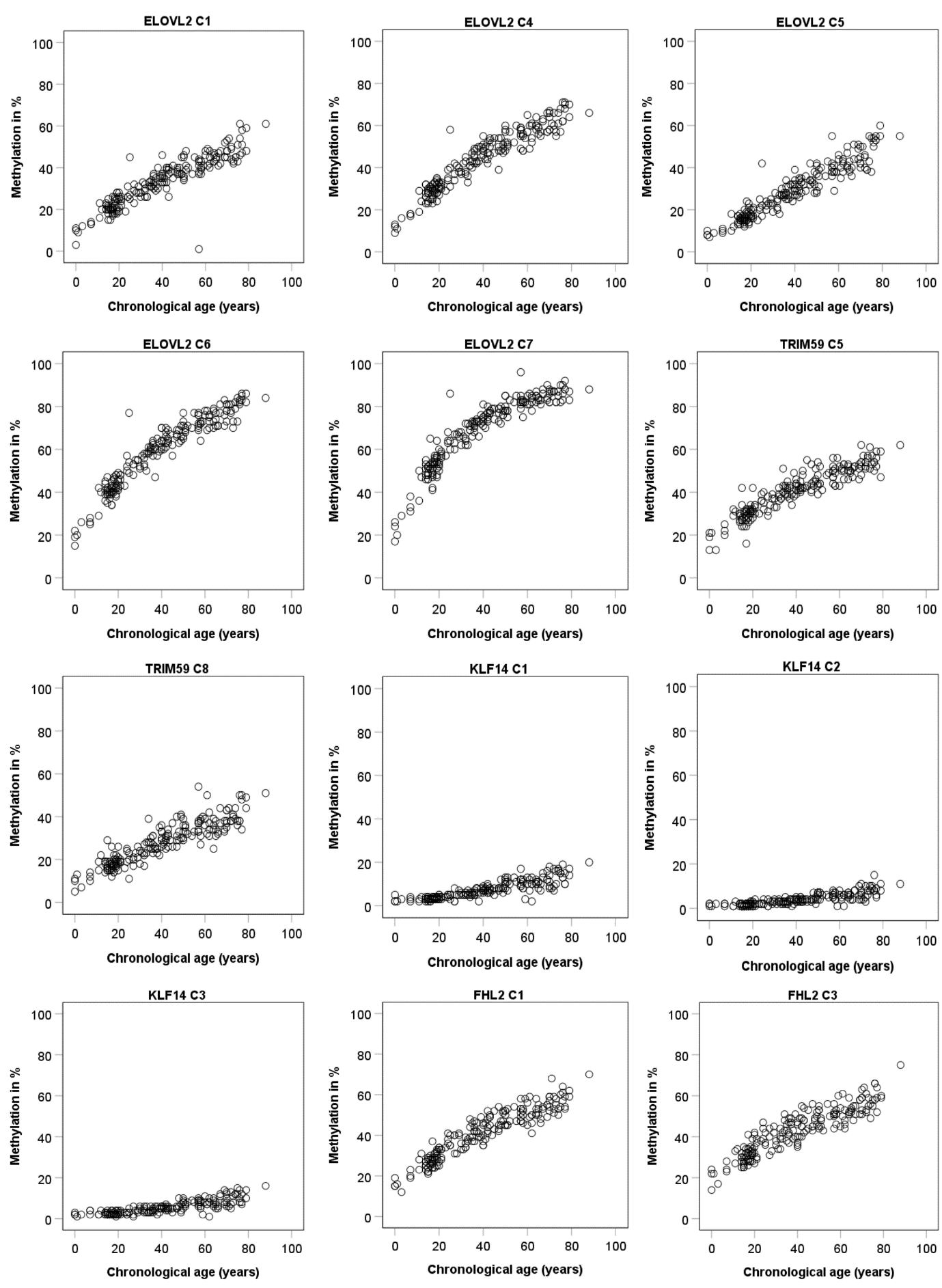


**Supplementary Figure S2**

Correlation between DNA methylation level and chronological age of selected CpGs in a training data set of 196 blood samples.


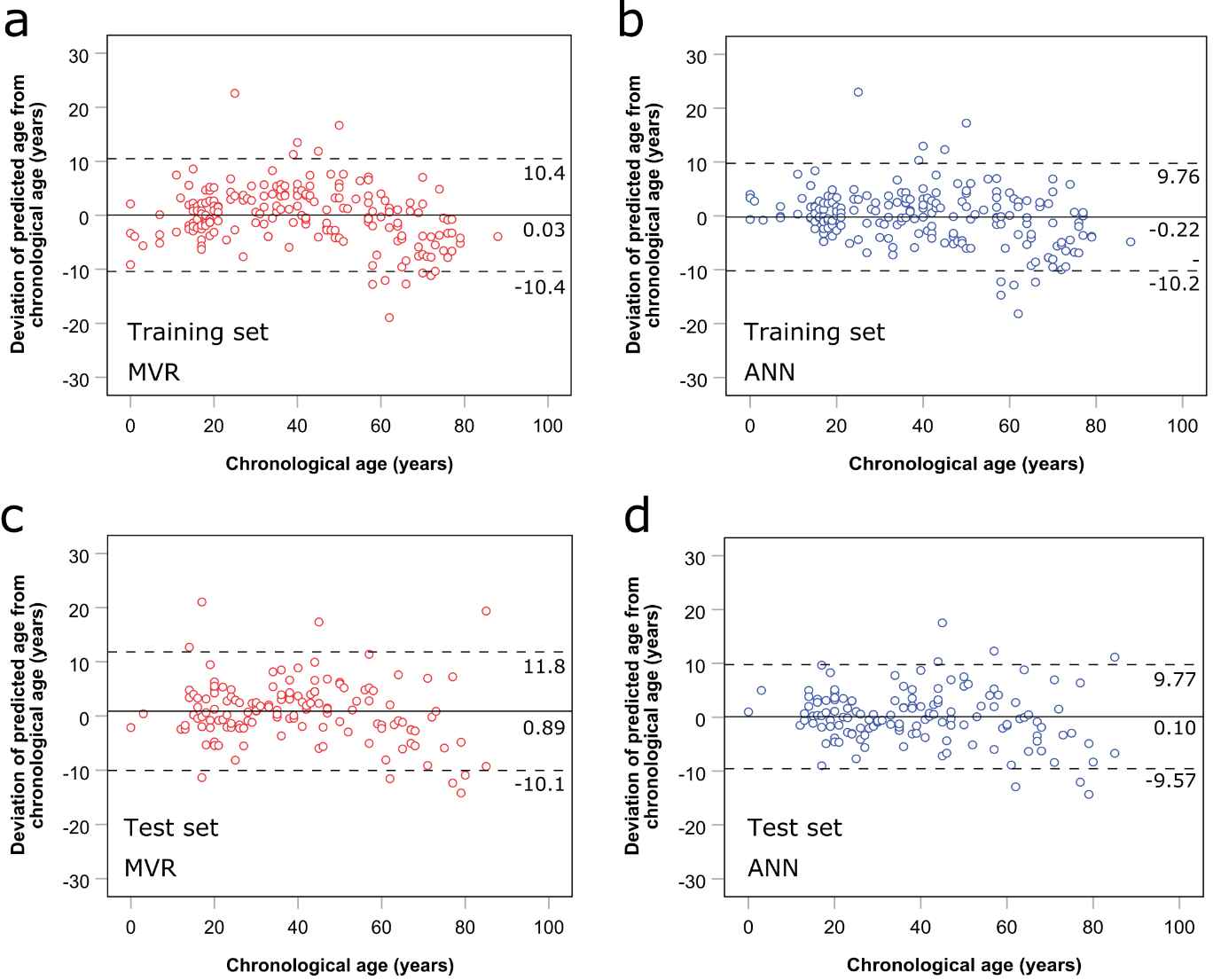


**Supplementary Figure S3**

Bland-Altman plots showing the deviations from chronological age against chronological age in (a, b) training (n = 196) and (c, d) test (n = 137) data sets. Values on the right of each Bland-Altman plot refer to mean (solid line) and 95% confidence interval (broken lines). MVR = multivariable regression; ANN = artificial neural network.


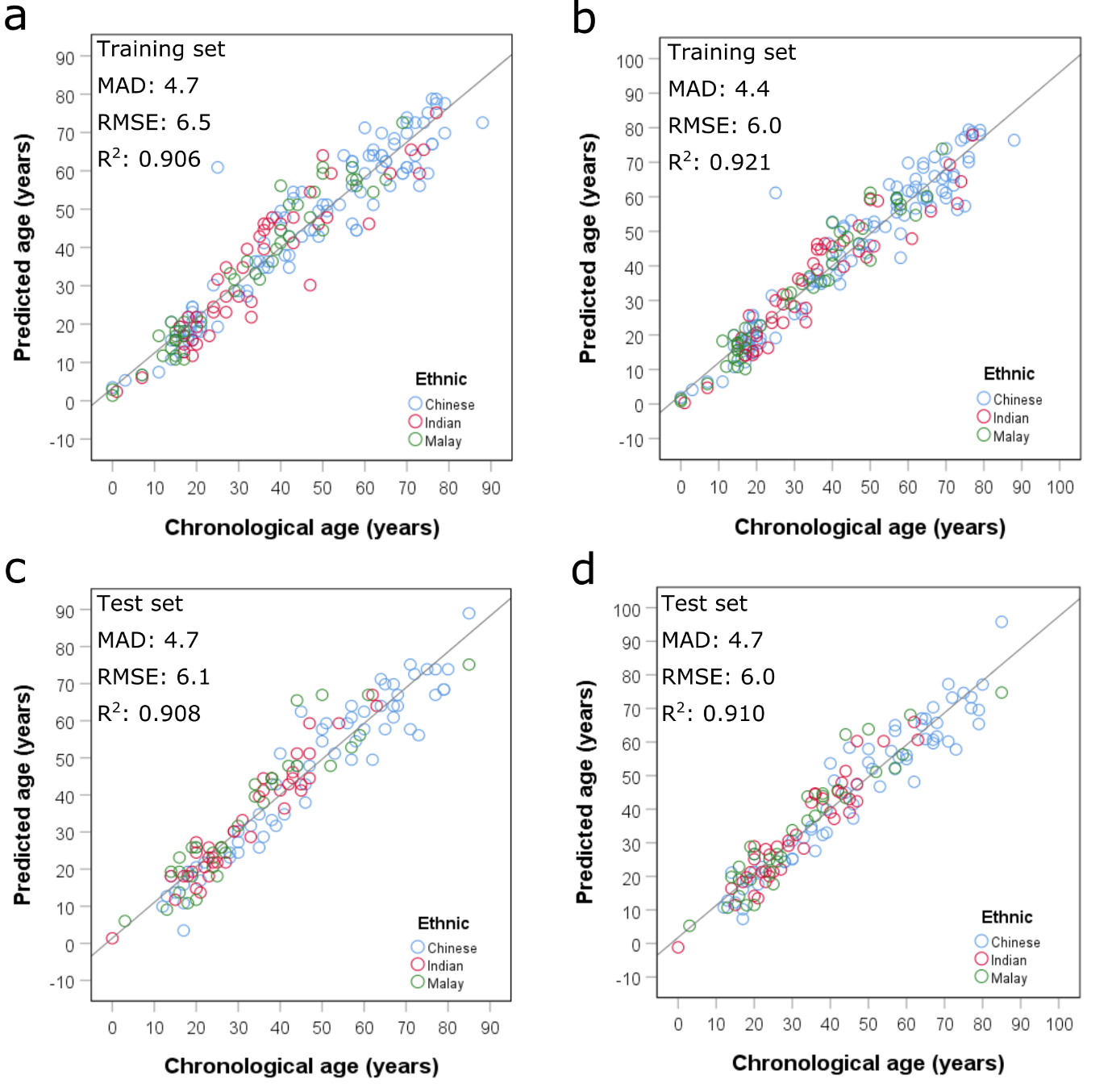


**Supplementary Figure S4**

Age prediction with sensitivity artificial neural network (ANN) models on (a, b) training (n =196) and (c, d) test (n = 137) data comprising the three local ethnic groups (Chinese, Indian, Malay). Age was predicted with (a, c) one predictor *ELOVL2* C4 and (b, d) two predictors *ELOVL2* C4 and *ELOVL2* C5. MAD = mean absolute deviation; RMSE = root mean square error. The units for MAD and RMSE are years.


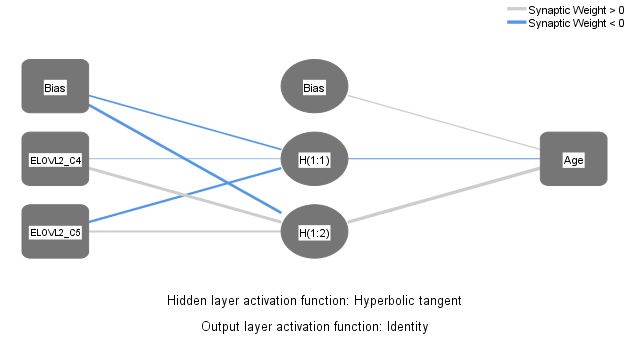


**Supplementary Figure S5**

Structural diagram of the dual CpG sensitivity artificial neural network (ANN) model. *ELOVL2* C4 and *ELOVL2* C5 are the input variables. Similar to the default ANN model in Supplementary Fig. S1, all weighted values passed to the hidden layer node are summed on the hidden layer node and passed to the output node through activation function using hyperbolic tangent. All weighted values entering the output node are summed again and passed through activation function using identity. The output values (predicted age) in the training of the ANN are compared with the chronological age of each sample. The weight is adjusted closer to the chronological age the next time the sample appears on the network.

**Supplementary Table S1** Primers used in this study were based on the Zbieć-Piekarska *et al*. study [1].

| Locus | Primers | Primer sequence | Primer concentration (µM) | Sequence to be analysed |
| --- | --- | --- | --- | --- |
| *ELOVL2* | Forward ^B^ | AGGGGAGTAGGGTAAGTGAGG | 0.2 | CCRTAAACRTTAAACCRCCRCRCRAAACCRAC |
|  | Reverse | AACAAAACCATTTCCCCCTAATAT | 0.2 |  |
|  | Sequencing | ACAACCAATAAATATTCCTAAAACT | 0.375 |  |
| *TRIM59* | Forward | TATAGGTGGTTTGGGGGAGAG | 0.2 | GGTTTGGYGYGGGAYGAGGYGAAGYGTYGGTGGTYGAYGG TTTTTGAGGAATTATTTTTTATTT |
|  | Reverse ^B^ | AAAAAACACTACCCTCCACAACATAAC | 0.2 |  |
|  | Sequencing | TTGGGGGAGAGGTTG | 0.375 |  |
| *KLF14* | Forward | GGTTTTTAGGTTAAGTTATGTTTAATAGT | 0.2 | TYGYGTTTTTTTTTTTGTYGGYGAGTTAGGTA ATGGTAATAGAG |
|  | Reverse ^B^ | ACTACTACAACCCAAAAATTCC | 0.2 |  |
|  | Sequencing | ATAGTTTTAGAAATTATTTTGTTT | 0.375 |  |
| *FHL2* | Forward | TGTTTTTAGGGTTTTGGGAGTATAG | 0.2 | AGTTATYGGGAGYGTYGTTTTYGGYGTGGGTTTTYGGGYG YGAGTTTYGGAYGAGGTTTGGG |
|  | Reverse ^B^ | ACACCTCCTAAAACTTCTCCAATCTCC | 0.2 |  |
|  | Sequencing | GGTTTTGGGAGTATAGT | 0.375 |  |

^B^ – Biotinylated PCR primer

Y – location of CpGs (pyrimidine C or T)

R – location of CpGs (purine A or G)

**Supplementary Table S2**

Network information.

| Input layer | Covariates | 1. ELOVL2 C4 |
| --- | --- | --- |
|  |  | 2. TRIM59 C5 |
|  |  | 3. KLF14_C1 |
|  | Number of units# | 3 |
|  | Rescaling method for covariates | Normalized |
| Hidden layer | Number of hidden layers | 1 |
|  | Number of units in hidden layer 1# | 2 |
|  | Activation function | Hyperbolic tangent |
| Output layer | Dependent variables | 1. Age |
|  | Number of units | 1 |
|  | Rescaling method for scale dependents | Normalized |
|  | Activation function | Identity |
|  | Error function | Sum of squares |

# excluding the bias unit

**Supplementary Table S3**

Parameter estimates of artificial neural network (ANN) model. The synaptic weights displays the coefficient estimates that show the relationship between the units in a given layer to the units in the following layer.

|  |  | Predicted | | |
| --- | --- | --- | --- | --- |
|  |  | Hidden layer 1 | | Output layer |
| Predictor | | H(1:1) | H(1:2) | Age |
| Input layer | (Bias) | -0.741 | 1.322 |  |
|  | ELOVL2 C4 | -0.277 | -0.967 |  |
|  | KLF14 C1 | 0.221 | -0.406 |  |
|  | TRIM59 C5 | -0.295 | -0.469 |  |
| Hidden layer 1 | (Bias) |  |  | -0.106 |
|  | H(1:1) |  |  | -1.081 |
|  | H(1:2) |  |  | -0.673 |

**Supplementary Table S4**

Reproducibility testing of pyrosequencing assays for 29 CpGs at four DNA methylation loci using ten samples. Methylation values refer to the average absolute difference in methylation between duplicates starting from bisulfite treatment stage.

| Locus | CpG | Absolute mean difference in DNA methylation (%) | Standard deviation (%) |
| --- | --- | --- | --- |
| *ELOVL2* | C1 | 2.7 | 1.9 |
|  | C2 | 2.0 | 1.5 |
|  | C3 | 3.0 | 1.9 |
|  | C4 | 3.0 | 2.0 |
|  | C5 | 2.6 | 1.9 |
|  | C6 | 2.2 | 2.1 |
|  | C7 | 2.5 | 1.7 |
| *KLF14* | C1 | 1.3 | 1.4 |
|  | C2 | 1.0 | 1.3 |
|  | C3 | 1.3 | 1.1 |
|  | C4 | 0.5 | 0.7 |
| *TRIM59* | C1 | 2.1 | 1.7 |
|  | C2 | 1.8 | 1.5 |
|  | C3 | 2.5 | 1.5 |
|  | C4 | 2.9 | 1.9 |
|  | C5 | 1.9 | 1.4 |
|  | C6 | 2.8 | 1.7 |
|  | C7 | 2.4 | 2.2 |
|  | C8 | 2.3 | 1.6 |
| *FHL2* | C1 | 1.8 | 1.3 |
|  | C2 | 1.8 | 1.5 |
|  | C3 | 1.8 | 1.5 |
|  | C4 | 1.3 | 1.0 |
|  | C5 | 2.0 | 1.4 |
|  | C6 | 1.6 | 1.8 |
|  | C7 | 1.4 | 1.1 |
|  | C8 | 2.1 | 1.6 |
|  | C9 | 2.1 | 1.6 |
|  | C10 | 1.9 | 1.3 |

**Supplementary Table S5**

Evaluation of the top five age prediction models generated by forward stepwise regression on 29 CpGs from *ELOVL2*, *KLF14*, *TRIM59* and *FHL2* on a training data set of 196 blood samples. Model highlighted in bold depicts the selected predictors used to build the multivariable regression and artificial neural network models. RMSE = root mean square error; MAD = mean absolute deviation; VIF = variance inflation factor.

| CpG | *R* | Adjusted *R*^2^ | 95% CI for *R*^2^ | RMSE | MAD | VIF | | | | |
| --- | --- | --- | --- | --- | --- | --- | --- | --- | --- | --- |
|  |  |  |  |  |  | *ELOVL2* C4 | *KLF14* C1 | *TRIM59* C5 | *ELOVL2* C5 | *ELOVL2* C1 |
| *ELOVL2* C4 | 0.947 | 0.896 | 0.869 ≤ *R*^2^ ≤ 0.923 | 6.9 | 5.2 | 1.000 | - | - | - | - |
| *ELOVL2* C4, *KLF14* C1 | 0.961 | 0.922 | 0.903 ≤ *R*^2^ ≤ 0.943 | 6.0 | 4.4 | 2.865 | 2.865 | - | - | - |
| ***ELOVL2* C4, *KLF14* C1, *TRIM59* C5** | **0.968** | **0.937** | **0.922 ≤ *R*^2^ ≤ 0.954#** | **5.4** | **4.1** | **5.957** | **3.037** | **5.443** | **-** | **-** |
| *ELOVL2* C4, *KLF14* C1, *TRIM59* C5, *ELOVL2* C5 | 0.971 | 0.941 | 0.927 ≤ *R*^2^ ≤ 0.957 | 5.2 | 3.9 | 11.692 | 3.460 | 5.697 | 12.610 | - |
| *ELOVL2* C4, *KLF14* C1, *TRIM59* C5, *ELOVL2* C5, *ELOVL2* C1 | 0.973 | 0.944 | 0.932 ≤ *R*^2^ ≤ 0.960 | 5.0 | 3.7 | 17.490 | 3.511 | 5.707 | 12.758 | 8.180 |

# 95% CI for ANN model comprising *ELOVL2* C4, *KLF14* C1 and *TRIM59* C5 is 0.928 ≤ *R*^2^ ≤ 0.958

**Supplementary Table S6**

Multivariable regression analysis (MVR) models adjusted for ethnicity, sex or both covariates using training (n = 196) and test (n = 137) data. The regression equations are as follows:

Ethnicity

Predicted age (years) = -25.581 + 0.763 x [*ELOVL2* C4] +1.155 x [*KLF14* C1] + 0.582 x [*TRIM59* C5] + 0.114 [Indian] - 0.855 [Malay]

Sex

Predicted age (years) = -26.332 + 0.772 x [*ELOVL2* C4] +1.181 x [*KLF14* C1] + 0.569 x [*TRIM59* C5] + 1.857 [Female]

Ethnicity & sex

Predicted age (years) = -25.864 + 0.775 x [*ELOVL2* C4] +1.174 x [*KLF14* C1] + 0.561 x [*TRIM59* C5] + 0.167 [Indian] - 1.033 [Malay] + 1.953 [Female]

Dummy variables

Indian: (1 = yes, 0 = no)

Malay: (1 = yes, 0 = no)

Female: (1 = yes, 0 = no)

Reference: Chinese, Male

|  | MAD (years) adjusted for covariate(s) | | |
| --- | --- | --- | --- |
| Data | Ethnicity | Sex | Ethnicity & sex |
| Training | 4.1 | 4.0 | 4.0 |
| Test | 4.2 | 4.1 | 4.1 |

**Supplementary Table S7**

Regression coefficient of the variable predictors *ELOVL2* C4, *KLF14* C1 and *TRIM59* C5 adjusted for ethnicity, sex or both covariates using training data (n = 196).

|  | Regression coefficient  (Absolute change in regression coefficient %) | | |
| --- | --- | --- | --- |
| Covariate | *ELOVL2* C4 | *KLF14* C1 | *TRIM59* C5 |
| - | 0.762  (1.7%) | 1.162  (1.0%) | 0.589  (5.0%) |
| Ethnicity | 0.763  (1.5%) | 1.155  (1.6%) | 0.582  (3.7%) |
| Sex | 0.772  (0.4%) | 1.181  (0.6%) | 0.569  (1.4%) |
| Ethnicity & sex | 0.775 | 1.174 | 0.561 |

**Supplementary Table S8**

Evaluation of the age prediction models generated by forward stepwise regression on seven CpGs from *ELOVL2* on a training data set of 196 blood samples. Model highlighted in bold depicts the selected predictors used to build the artificial neural network model. RMSE = root mean square error; VIF = variance inflation factor.

| CpG | *R* | Adjusted *R*^2^ | 95% CI for *R*^2^ | RMSE | VIF | | |
| --- | --- | --- | --- | --- | --- | --- | --- |
|  |  |  |  |  | *ELOVL2* C4 | *ELOVL2* C5 | *ELOVL2* C1 |
| ***ELOVL2* C4** | **0.947** | **0.896** | **0.869 ≤ *R*^2^ ≤ 0.923#** | **6.9** | **1.000** |  |  |
| ***ELOVL2* C4, *ELOVL2* C5** | **0.958** | **0.918** | **0.896 ≤ *R*^2^ ≤ 0.940^** | **6.1** | **10.185** | **10.185** |  |
| *ELOVL2* C4, *ELOVL2* C5, *ELOVL2* C1 | 0.960 | 0.921 | 0.900 ≤ *R*^2^ ≤ 0.942 | 6.1 | 16.021 | 10.249 | 8.057 |

# 95% CI for ANN model comprising *ELOVL2* C4 is 0.881 ≤ *R*^2^ ≤ 0.931

^ 95% CI for ANN model comprising *ELOVL2* C4 and C5 is 0.900 ≤ *R*^2^ ≤ 0.942

**Supplementary Table S9**

Parameter estimates of dual CpG sensitivity artificial neural network (ANN) model. The synaptic weights displays the coefficient estimates that show the relationship between the units in a given layer to the units in the following layer.

|  |  | Predicted | | |
| --- | --- | --- | --- | --- |
|  |  | Hidden layer 1 | | Output layer |
| Predictor | | H(1:1) | H(1:2) | Age |
| Input layer | (Bias) | -0.539 | -0.813 |  |
|  | ELOVL2 C4 | -0.131 | 0.833 |  |
|  | ELOVL2 C5 | -0.588 | 0.557 |  |
| Hidden layer 1 | (Bias) |  |  | 0.486 |
|  | H(1:1) |  |  | -0.149 |
|  | H(1:2) |  |  | 0.836 |

**Supplementary Table S10**

MADs and percentage of correct predictions of different models on French individuals (n = 100). MVR 1 refers to the multivariable regression model developed in our previous study [2]. The results of MAD and correct prediction were previously reported by Daunay *et al* [3]. MVR 2 and ANN refers to the multivariable regression and artificial neural network models developed in our current study. MAD = mean absolute deviation. Correct prediction is calculated based on ≤ 5 years between predicted and actual ages.

| Model | MAD (years) | Correct prediction % (n) |
| --- | --- | --- |
| MVR 1 | 5.2 | 55 (55/100) |
| MVR 2 | 5.0 | 61 (61/100) |
| ANN | 4.8 | 68 (68/100) |

**Supplementary Table S11**

Convicted penal population as of 31 December 2018. Raw data were obtained from the Singapore Prison Service Annual Statistics 2018 and subsequently calculated based on modified age ranges in each age group.

|  | Proportion of convicted penal inmates % (n) | | |
| --- | --- | --- | --- |
| Age group | 2016 | 2017 | 2018 |
| Below 21 | 3 (278) | 3 (227) | 3 (235) |
| 21-40 | 37 (3503) | 37 (3297) | 38 (3322) |
| 41-60 | 54 (5140) | 53 (4688) | 51 (4443) |
| 61 and above | 6 (581) | 8 (673) | 9 (781) |
| Overall | 100 (9502) | 100 (8885) | 100 (8781) |

**Reference**

[1] Zbieć-Piekarska, R. *et al*. Development of a forensically useful age prediction method based on DNA methylation analysis. *Forensic Sci Int Genet* **17**, 173 – 179 (2015).

[2] Thong, Z., Chan, X. L. S.,Tan, J. Y. Y. , Loo, E. S. & Syn, C. K. C. Evaluation of DNA methylation-based age prediction on blood. *Forensic Sci Int Genet Suppl Ser* **6**, e249 – 251 (2017).

[3] Daunay, A., Baudrin L. G., Deleuze, J. F. & How-Kit, A. Evaluation of six blood-based age prediction models using DNA methylation analysis by pyrosequencing. *Sci Rep* **9**, 8862 (2019).
