# Supplementary material for: Artificial neural network, predictor variables and sensitivity threshold for DNA methylation-based age prediction using blood samples
Source: Sci Rep. 2021 Jan 18;11:1744. doi: 10.1038/s41598-021-81556-2 (PMC7814006; doi:10.1038/s41598-021-81556-2)
Supplement: Supplementary file 6 — Supplementary Information. [file 41598_2021_81556_MOESM6_ESM.docx]

**Steps to use age prediction ANN model to predict the age of an individual from an unknown blood sample**

1. Enter the methylation values (%) for *ELOVL2* C4, *TRIM59* C5 and *KLF14* C1 in the “Input template default”.xlsx and save the file
2. Open the saved file using IBM SPSS statistics software
3. Under “Utilities”, select “Scoring Wizard” and click “Browse” to locate the “ANN age prediction calculator default” and select it
4. On the “Scoring Wizard” window, press “Next >” and ensure the CpG predictors in the “Dataset Fields” match those in “Model Fields”. Otherwise, manually change CpG predictors in the “Dataset Fields”
5. On the “Scoring Wizard” window, click “Finish”
6. The predicted age is calculated and presented under the column “PredictedValue”

**Steps to use age prediction ANN sensitivity model to predict the age of an individual from an unknown blood sample**

1. Enter the methylation values (%) for *ELOVL2* C4 and *ELOVL2* C5 in the “Input template sensitivity”.xlsx and save the file
2. Open the saved file using IBM SPSS statistics software
3. Under “Utilities”, select “Scoring Wizard” and click “Browse” to locate the “ANN age prediction calculator sensitivity” and select it
4. On the “Scoring Wizard” window, press “Next >” and ensure the CpG predictors in the “Dataset Fields” match those in “Model Fields”. Otherwise, manually change CpG predictors in the “Dataset Fields”
5. On the “Scoring Wizard” window, click “Finish”
6. The predicted age is calculated and presented under the column “PredictedValue”
